# A systematic review and meta-analysis comparing outcomes following total knee arthroplasty for rheumatoid arthritis versus for osteoarthritis

**DOI:** 10.1186/s12891-023-06601-9

**Published:** 2023-06-13

**Authors:** Yongjie Qiao, Feng Li, Lvdan Zhang, Xiaoyang Song, Xinyuan Yu, Haoqiang Zhang, Peng Liu, Shenghu Zhou

**Affiliations:** 1 Department of Joint Surgery, The 940th Hospital of Joint Logistic Support Force of Chinese People’s Liberation Army, Gansu, Lanzhou China; 2 Department of Orthopedics, The 943rd Hospital of Joint Logistic Support Force of Chinese People’s Liberation Army, Gansu, Wuwei China; 3 Department of Respiratory Medicine, The 940th Hospital of Joint Logistic Support Force of Chinese People’s Liberation Army, Gansu, Lanzhou China

**Keywords:** Rheumatoid arthritis, Osteoarthritis, Complications, Outcomes, Total knee arthroplasty, Meta-analysis

## Abstract

**Purpose:**

Total knee arthroplasty (TKA) in patients with osteoarthritis (OA) are considered to be a successful procedure, but with little being known about outcomes in patients with rheumatoid arthritis (RA). The aim of this study was to compare the outcomes of TKA in patients with RA versus OA.

**Methods:**

Data were obtained from PubMed, Cochrane Library, EBSCO and Scopus for all available studies comparing the outcomes of THA in RA and OA patients (From January 1, 2000 to October 15, 2022). Outcomes of interest included infection, revision, venous thromboembolism (VTE), mortality, periprosthetic fractures, prosthetic loosening, length of stay, and satisfaction. Two reviewers independently assessed each study for quality and extracted data. The quality of the studies was scored using the Newcastle-Ottawa scale (NOS).

**Results:**

Twenty-four articles with a total 8,033,554 patients were included in this review. The results found strong evidence for increased risk of overall infection (OR = 1.61, 95% CI, 1.24–2.07; *P* = 0.0003), deep infection (OR = 2.06, 95% CI, 1.37–3.09; *P* = 0.0005), VTE (OR = 0.76, 95% CI, 0.61–0.93; *P* = 0.008), pulmonary embolism (PE) (OR = 0.84, 95% CI, 0.78–0.90; *P*<0.00001), periprosthetic fractures (OR = 1.87, 95% CI, 1.60–2.17; *P*<0.00001); and reasonable evidence for increased risk of deep venous thrombosis (DVT) (OR = 0.74, 95% CI, 0.54–0.99; *P* = 0.05), and length of stay (OR = 0.07, 95% CI, 0.01–0.14; *P* = 0.03) after TKA in patients with RA versus OA. There were no significant differences in superficial site infection (OR = 0.84,95% CI, 0.47–1.52; *P* = 0.57), revision (OR = 1.33,95% CI, 0.79–2.23; *P* = 0.28), mortality (OR = 1.16,95% CI, 0.87–1.55; *P* = 0.32), and prosthetic loosening (OR = 1.75, 95% CI, 0.56–5.48; *P* = 0.34) between the groups.

**Conclusion:**

Our study demonstrated that patients with RA have a higher risk of postoperative infection, VTE, periprosthetic fracture, and lengths of stay, but did not increase revision rate, prosthetic loosening and mortality compared to patients with OA following TKA. In conclusion, despite RA increased incidence of postoperative complications, TKA should continue to be presented as an effective surgical procedure for patients whose conditions are intractable to conservative and medical management of RA.

**Supplementary Information:**

The online version contains supplementary material available at 10.1186/s12891-023-06601-9.

## Introduction

Total knee arthroplasty (TKA) is considered one of the most utilized and successful procedures available to resolve end-stage knee disease and can significantly improve patients’ quality of life and knee function postoperatively [1, 2]. With progressive global aging, the annual worldwide rate of TKA has increased steadily over the past two decades [3]. The main cause of end-stage arthritis is osteoarthritis (OA), which accounts for 90–97% of the primary indication for TKA, followed by rheumatoid arthritis (RA) [4, 5]. Outcomes following TKA are generally unexceptionable, with low complication rate and high satisfaction [6]. However, some complications including infection, revision, venous thromboembolism (VTE), even death have been troubling surgeons significantly [7–9].

RA is a chronic, symmetrical, progressive, inflammatory autoimmune disease that primarily affects the joints and is characterized by symmetrical, multi-articular, invasive joint inflammation of the joints of the whole body, especially the hands and feet, leads to the degeneration of cartilage and destruction of bones and joint structure eventually [10]. Patients with RA reported can be significantly improved in pain and function after TKA, yet major outcomes such as infection, revision, and readmission are reported to be higher for patients with RA compared to patients with OA [11, 12]. Since the utilization of disease modifying antirheumatic drugs (DMARDs), biologic agents and Janus kinase (JAK) inhibitors widely have been dramatically enhanced the quality of life for patients with RA, so the number of TKA has been declining in recent years [13, 14]. However, patients with RA may develop osteoporosis and ligament relaxation with joint deformity and disability from controlled RA unsatisfactorily, therefore, operation is still a crucial option for RA treatment [15].

While most TKA operations are performed in patients with OA, which is the most common form of arthritis, they are also efficient in treating progressive joint destruction of the knee in patients with RA [6, 16]. Therefore, numerous previous studies with respect to outcomes of TKA is relied on the experience in patients with OA [3, 17]. Both patients with RA and OA can be treated with TKA, but RA is essentially different from OA in terms of pathogenesis, prognosis, and medical therapy, so conceivable differences in TKA outcomes would be expected [6, 18].

However, few studies have inquired whether there are differences in outcomes for patients with RA versus OA. Some previous meta-analyses have demonstrated that TKA can improve the outcomes of patients with RA, but compared with OA, patients with RA are at higher risk of complications after TKA [6, 19]. However, these studies analyzed a few outcomes, and some literatures were older, and some literatures did not set control group, so the results obtained are controversial. Therefore, our current study was designed to compared outcomes following TKA for RA Versus for OA by pooling data from previous comparative studies (From January 1, 2000 to October 15, 2022). It was hypothesized that the incidence of infection, revision, VTE, mortality, periprosthetic fractures, prosthetic loosening and length of stay all would be higher, and lower satisfaction after TKA in patients with RA than in patients with OA.

## Materials and methods

### Data and literature sources

This systematic review and meta-analysis adhered to the Preferred Reporting Items for Systematic Reviews and Meta-Analyses (PRISMA) guidelines. We performed a systematic search of various electronic databases (i.e.: PubMed, Cochrane Library, EBSCO and Scopus), including reports published from January 1, 2000 to October 15, 2022 (to more closely reflect current clinical practice) that described studies of primary TKA and contained information on outcomes in RA and OA patients, without language and date restrictions. Broad MeSH terms and Boolean operators were selected for each database search; the following search terms were used: (Total knee arthroplasty OR TKA OR total knee replacement OR TKR) and (rheumatoid arthritis OR RA) and (Osteoarthritis OR OA). All obtained by searching titles and abstracts were carefully evaluated, and then full texts were read to determine the included articles.

### Study selection

#### Inclusion and exclusion criteria

Two authors independently selected abstracts as well as full-text articles from the above listed databases using the aforementioned search strategies, and a third author adjudicated discrepancies.

#### The inclusion criteria were listed as follows

(1) Case-control studies (CCS), retrospective cohort studies (RCS), or prospective cohort studies (PCS) comparing outcomes in patients with RA and OA undergoing primary TKA were included; (2) at least one of the following outcome measures was reported: infection (periprosthetic joint infection or wound infection), revision, VTE (deep venous thrombosis (DVT) or pulmonary embolism (PE)), mortality, periprosthetic fractures, prosthetic loosening, length of stay, and satisfaction; (3) without restrictions on age and sex were imposed; and (4) without limitations on language and race were imposed.

#### The following exclusion criteria were used

(1) Non-peer reviewed publications; (2) certain study designs (non-human trials, observational studies, case reports, case series, review articles, letters to the editor); (3) the inclusion and exclusion criteria for the study were not clear or reasonable; and (4) the full text cannot be obtained or the original data are incomplete.

### Data extraction

The following data were extracted: (1) demographic and clinical information of the studies(including author, year of publication, country, study type, study period, sample size, follow-up); (2) outcome measures: including infection, revision, VTE, DVT, PE, mortality, periprosthetic fractures, prosthetic loosening, length of stay and satisfaction. Pertinent data were extracted by two reviewers independently from all eligible studies in patients with RA in comparison to patients with OA using a standardized data collection form, and any disagreement was resolved by a third reviewer.

### Assessment of methodological quality

For each included study, the methodological quality was evaluated using Newcastle-Ottawa scale (NOS) [20] by two independent reviewers. NOS Scale is a tool for quality assessment of case-control studies and cohort studies. The domains included case definition (selection of study cohorts, comparability of the cohorts, and outcome ascertainment). The total scores were 9, it had high quality when NOS scores ≥ 6.

### Statistical analysis

All analyses were conducted using the RevMan software (RevMan version 5.3.5, The Nordic Cochrane Centre, The Cochrane Collaboration 2014, Copenhagen, Denmark). For continuous variables, this software estimates the weighted mean differences (WMD). The Mantel-Haenszel model and odds ratios (ORs) with 95% confidence intervals (CIs) for outcomes of interest were used to compare dichotomous variables. The effect size (ES) was used for analysis when the units of odds ratios (ORs) with 95% confidence intervals (CIs) were consistent. A *P*-value less than 0.05 was considered statistically significant. We calculated the *I*^2^ coefficient to assess heterogeneity with the following predetermined limits: low < 50%, moderate 50–74%, and high > 75%; and *P* ≥ 0.05 and *I*^2^ < 50% indicating no statistical heterogeneity between studies. A random-effects model was applied in circumstances of moderate or high heterogeneity; otherwise, a fixed-effects model was employed. If there was significant heterogeneity in the included studies, such data were considered unsuitable. Sensitivity analysis was conducted to assess the stability of the results if necessary. If there have other available data, subgroup analysis was also conducted to get more specific conclusions. Moreover, using the forest plots to depict the results of each study and evaluate pooled estimates respectively, and the funnel plots were used to evaluate publication bias.

## Results

### Study selection and quality of included studies

**Fig. 1:**
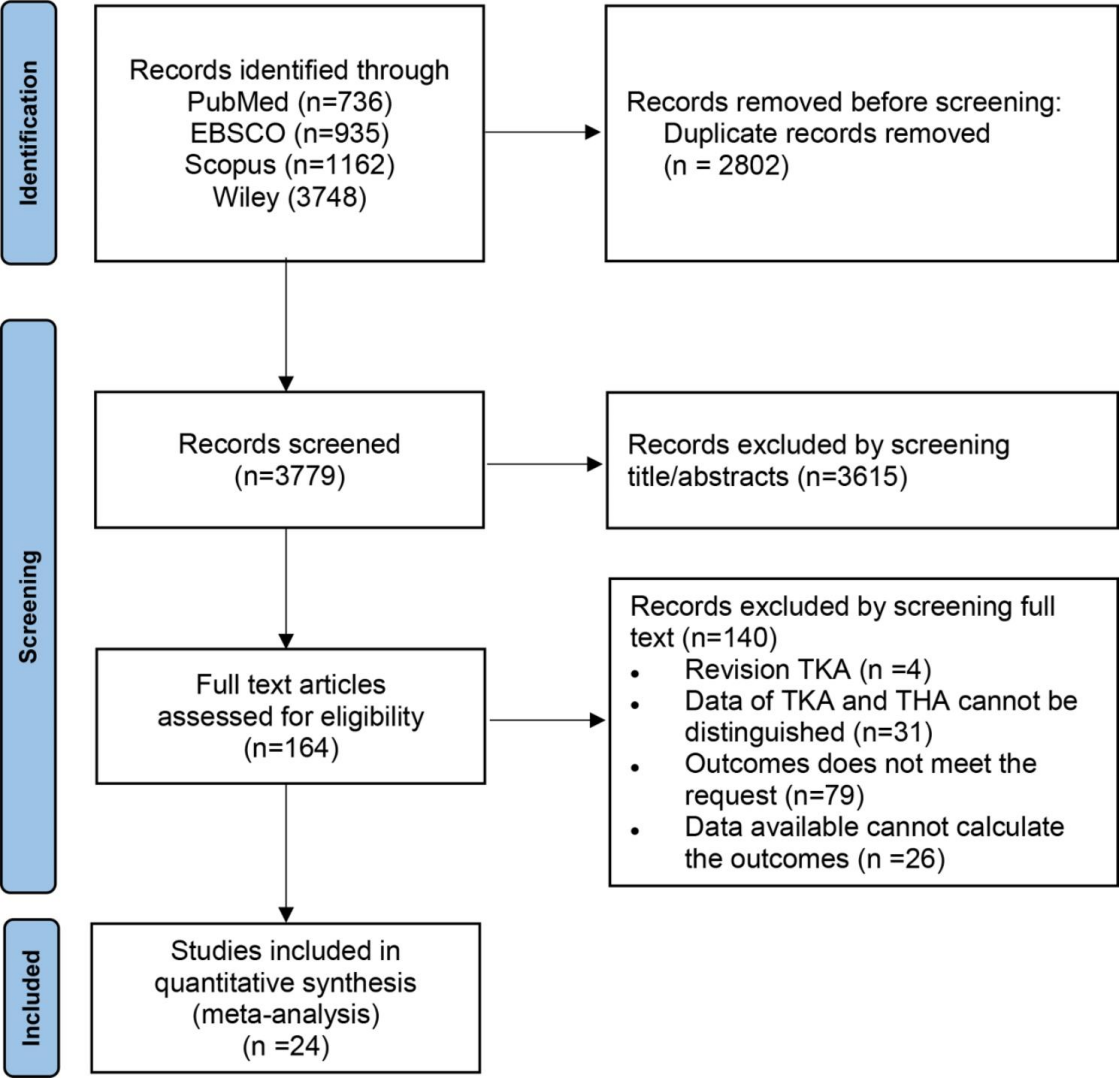
PRISMA flow diagram of the identification and selection of the studies included in this meta-analysis

The search strategy previously described produced 6581 results (736 in PubMed, 935 in EBSCO, 1162 in Scopus and 3748 in Cochrane Library). 2802 duplicates were deleted by citation management software and manual review of records. After reviewed the titles and abstracts by two independent authors, 3615 irrelevant citations were removed. The remaining 164 full text papers were then retrieved for a more detailed analysis, of which 140 papers were excluded for several reasons, such as revision TKA (n = 4), data of TKA and THA cannot be distinguished (n = 31), outcomes does not meet the request (n = 79), data available cannot calculate the outcomes (n = 26). Finally, 24 studies were included in our study and could be quantitatively synthesized and the remaining two were qualitatively analyzed. The article selection process is illustrated in Fig. 1.

**Fig. 2:**
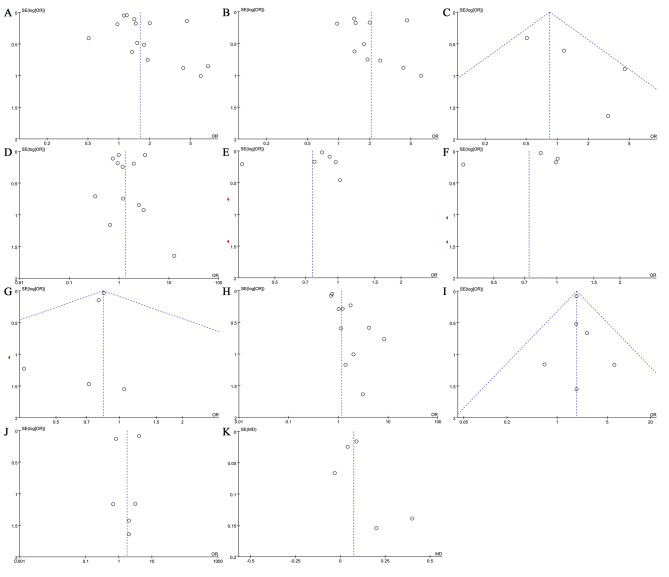
Funnel plots were used to evaluate publication bias. (**A**) overall infection (**B**) deep infection (**C**) superficial infection (**D**) revision (**E**) VTE (**F**) DVT (**G**) PE (**H**) mortality (**I**) periprosthetic fractures (**J**) prosthetic loosening

All studies [7–9, 11, 18, 21–39] including 15 RCS, 7 PCS, and 2 CCS had high quality with NOS scores ≥ 6 involved 7,786,321 patients in the OA group and 247,233 patients in the RA group. The quality evaluation and the basic characteristics of the selected trials are shown in Table 1. Outcomes after primary TKA in patients with RA versus patients with OA are shown in Table 2. Funnel plots were assessed for the potential publication bias, according to the funnel plot (Fig. 2), the influence of publication bias on the results could be ignored.

**Table 1:** Characteristics of the included studies

First Author	Year	Country	Study period	Study type	Sample size		Age(years)		Follow-up	NOS score	Outcome measures
					RA	OA	RA	OA			
*Baek JH* [11]	2022	South Korea	2007–2009	CCS	57	114	60.2 ^**a**^	60.3 ^**a**^	10 years ^**c**^	8	IN, RE, PPF, PL, MO
*Chung HK* [7]	2021	China	2012–2015	RCS	1126	63,215	64.8 ^**a**^	70.9 ^**a**^	3 months	7	IN, LOS
*Li Z* [8]	*2020*	China	1993–2017	RCS	138	1299	64.9 ^**a**^	64.9 ^**a**^	6.5 years ^**a**^	8	IN, VTE, DVT, PE, PPF, PL
*Mooney L* [9]	*2019*	Australia	2003–2016	RCS	7542	534,202	64.5 ^**a**^	68.6 ^**a**^	NA	6	IN, RE, PL, MO
*Blevins JL* [21]	*2019*	US	2007–2010	RCS	76	152	64.3 ^**a**^	64.5 ^**a**^	2 years ^**c**^	8	RE, PL, LOS
*Kobayashi S* [30]	*2019*	Japan	NA	RCS	75	459	66.2 ^**a**^	72.8 ^**a**^	2 years ^**c**^	7	IN, RE, PL
*Sajjadi MM* [18]	2019	Iran	2013–2014	PCS	33	138	58.1 ^**a**^	69.4 ^**a**^	12 months	7	IN, RE, MO
*Burn E* [23]	2018	Spain	1995–2014	RCS	639	10,322	70.0 ^**b**^	70.0 ^**b**^	3 months ^**c**^	6	IN, RE, VTE, MO
*Tayton ER* [39]	*2016*	New Zealand	1999–2012	RCS	2148	60,787	NA	NA	1 year	6	RE
*Goodman SM* [26]	*2016*	US	2007–2010	RCS	136	4320	63.5 ^**a**^	67.2 ^**a**^	2 years	8	LOS
*Schnaser EA* [36]	*2015*	US	2002–2011	RCS	209,916	6,616,985	64.0 ^**a**^	66.0 ^**a**^	NA	6	IN, VTE, DVT, PE, PPF, MO
*LoVerde ZJ* [31]	2015	US	2007–2010	CCS	159	318	63.6 ^**a**^	63.8 ^**a**^	6months ^**c**^	8	IN, RE, VTE, DVT, PE, PPF
*Izumi M* [28]	*2015*	Japan	2007–2010	PCS	204	1084	67.5 ^**a**^	75.0 ^**a**^	1 month	8	VTE, DVT, PE
*Ravi B* [34]	*2014*	Canada	2002–2009	RCS	2692	59,564	66.0 ^**b**^	68.0 ^**b**^	2 years ^**c**^	8	IN, RE, VTE, PPF, MO
*Stundner O* [38]	2014	US	2006–2010	RCS	11,755	339,348	64.3 ^**a**^	66.6 ^**a**^	NA	7	IN, VTE, DVT, PE, LOS, MO
*da Cunha BM* [25]	*2011*	Brazil	1996–2007	RCS	28	56	54.9 ^**a**^	71.0 ^**a**^	11 years	7	IN
*Schrama JC* [37]	*2010*	Norway	1994–2008	PCS	2462	21,832	64.0 ^**a**^	71.0 ^**a**^	6 years ^**c**^	8	RE
*Niki Y* [32]	2010	Japan	2003–2007	PCS	238	169	59.9 ^**a**^	74.2 ^**a**^	3 months ^**c**^	6	VTE, DVT, PE
*Jämsen E* [29]	2009	Finland	1997–2004	RCS	3040	35,298	71.0 ^**b**^	71.0 ^**b**^	3 years ^**b**^	6	IN
*Chesney D* [24]	2008	UK	1998–2005	PCS	71	1235	NA	NA	6 months ^**c**^	7	IN
*Himanen AK* [27]	*2005*	Finland	1985–1999	RCS	2161	6306	61.0 ^**a**^	70.0 ^**a**^	3.2 years ^**c**^	6	RE
*Ohzawa S* [33]	*2001*	Japan	1989–1996	RCS	81	53	62.6 ^**a**^	68.4 ^**a**^	5.4 years ^**a**^	7	MO
*Robertsson O* [35]	2001	Sweden	1988–1997	PCS	4495	35,163	66.0 ^**a**^	72.0 ^**a**^	3.8 years ^**b**^	8	IN, RE, PPF, PL
*Böhm P* [22]	2000	Germany	1972–1994	PCS	122	208	66.0 ^**a**^	72.0 ^**a**^	6 years ^**a**^	7	IN, MO

**Abbreviations:** RA, rheumatoid arthritis; OA, osteoarthritis; NOS, Newcastle-Ottawa Scale; CCS, case-control studies; RCS, retrospective cohort studies; PCS, prospective cohort studies; NA, not acquire; IN, infection; RE, revision; VTE, Venous thromboembolism; DVT, deep venous thrombosis; PE, pulmonary embolism; PPF, periprosthetic fractures; PL, prosthetic loosening; LOS, length of stay; MO, mortality

**Note:**^**a**^ Values are expressed as mean; ^**b**^ Values are expressed as median; ^**c**^ Values are expressed as minimum

**Table 2:** Outcomes after primary TKA in patients with RA versus patients with OA

Outcome or Subgroup	Studies	Participants		Statistical Method	I^2^	Effect Estimate	P value
		RA	OA				
All infection	16	241,888	7,697,924	OR (M-H, Random, 95% CI)	88%	1.61 [1.24, 2.07]	**0.0003**
Deep infection	13	20,058	741,273	OR (M-H, Random, 95% CI)	83%	2.06 [1.37, 3.09]	**0.0005**
Superficial infection	4	396	2908	OR (M-H, Fixed, 95% CI)	49%	0.84 [0.47, 1.52]	0.57
Revision	12	22,539	729,357	OR (M-H, Random, 95% CI)	96%	1.33 [0.79, 2.23]	0.28
VTE	8	225,741	7,029,089	OR (M-H, Random, 95% CI)	69%	0.76 [0.61, 0.93]	**0.008**
DVT	6	222,410	6,959,203	OR (M-H, Random, 95% CI)	78%	0.74 [0.54, 0.99]	**0.05**
PE	6	222,410	6,959,203	OR (M-H, Fixed, 95% CI)	0%	0.84 [0.78, 0.90]	**<0.00001**
Mortality	11	230,637	7,404,550	OR (M-H, Random, 95% CI)	72%	1.16 [0.87, 1.55]	0.32
Periprosthetic fractures	6	217,457	6,713,443	OR (M-H, Fixed, 95% CI)	0%	1.87 [1.60, 2.17]	**<0.00001**
Prosthetic loosening	6	12,383	571,389	OR (M-H, Random, 95% CI)	95%	1.75 [0.56, 5.48]	0.34
Length of stay	5	13,252	407,353	WMD (IV, Random, 95% CI)	65%	0.07 [0.01, 0.14]	**0.03**

**Abbreviations:** TKA, total knee arthroplasty; RA, rheumatoid arthritis; OA, osteoarthritis; OR, odds ratio; M-H, Mantel-Haenszel; CI, confidence interval; VTE, venous thromboembolism; DVT, deep venous thrombosis; PE, pulmonary embolism; WMD, weighted mean difference; IV, inverse variance

### Outcomes of the meta-analysis

#### Infection

**Fig. 3:**
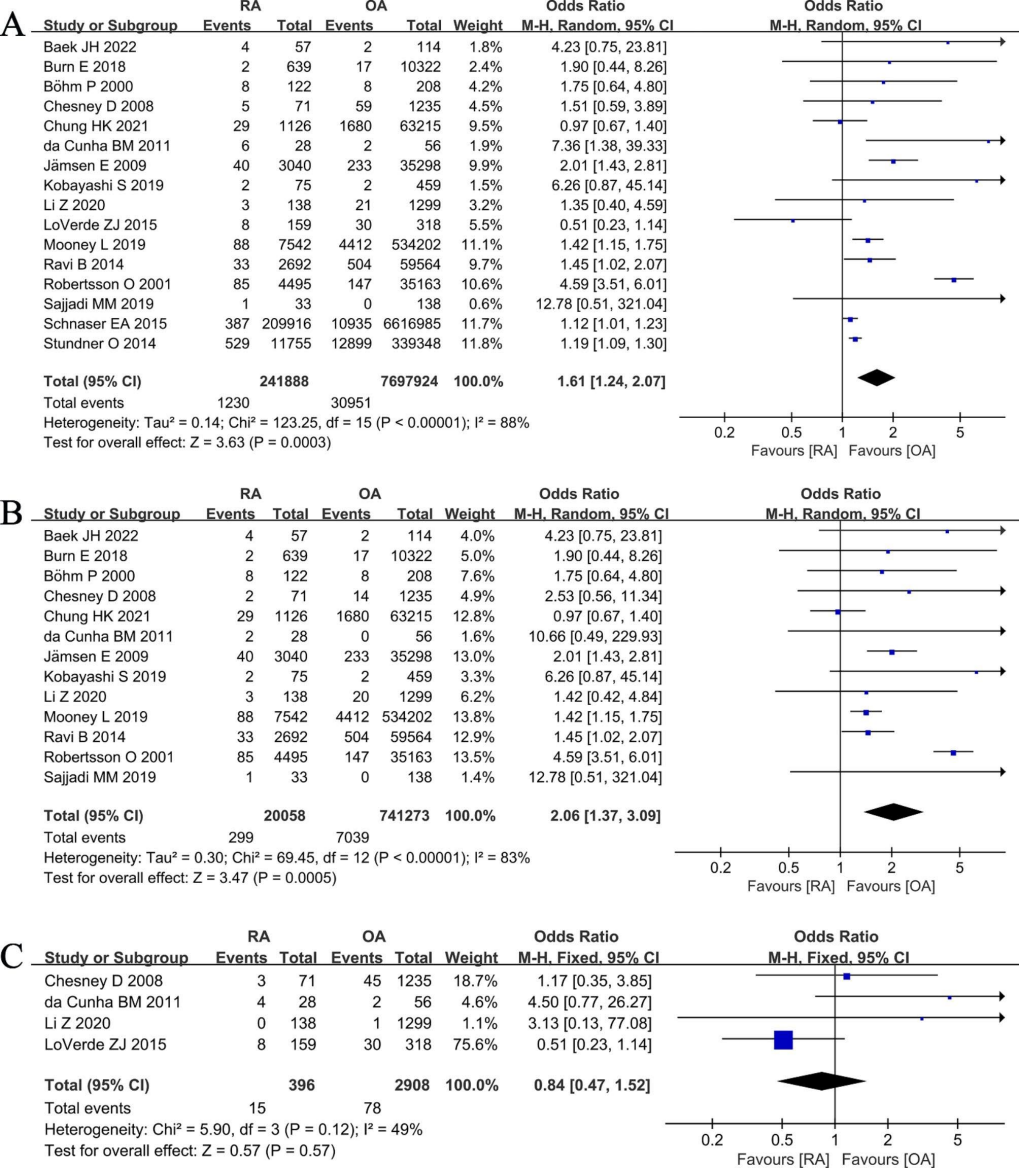
Forest plot showing the infection rate after primary TKA in patients with RA versus patients with osteoarthritis OA. (**A**). overall infection (**B**). deep infection (**C**). superficial infection

Sixteen [7–9, 11, 18, 22–25, 29–31, 34–36, 38] studies compared the rate of postoperative infection in patients with RA versus OA who underwent primary TKA. Meta-analysis of these 16 studies showed that the rate of postoperative infection was significantly higher in RA group than that in OA group (1230/241,888 vs. 30,951/7,697,924; OR 1.61, [95% CI 1.24–2.07]; *I*^2^ = 88%; *P* = 0.0003) (Fig. 3A). We performed a subgroup analysis based on deep and superficial infections. Subgroup analysis of 13 studies [7–9, 11, 18, 22–25, 29, 30, 34, 35] reported that the rate of deep infection after TKA was significantly higher in RA group than that in OA group (299/20,058 vs. 7039/741,273; OR 2.06, [95% CI 1.37–3.09]; *I*^2^ = 83%; *P* = 0.0005) (Fig. 3B). However, subgroup analysis of 4 studies [8, 24, 25, 31] reported that the rate of superficial infection were similar in RA and OA groups (15/396 vs. 78/2908; OR 0.84, [95% CI 0.47–1.52]; *I*^2^ = 49%; *P* = 0.57) (Fig. 3C).

#### Revision

**Fig. 4:**
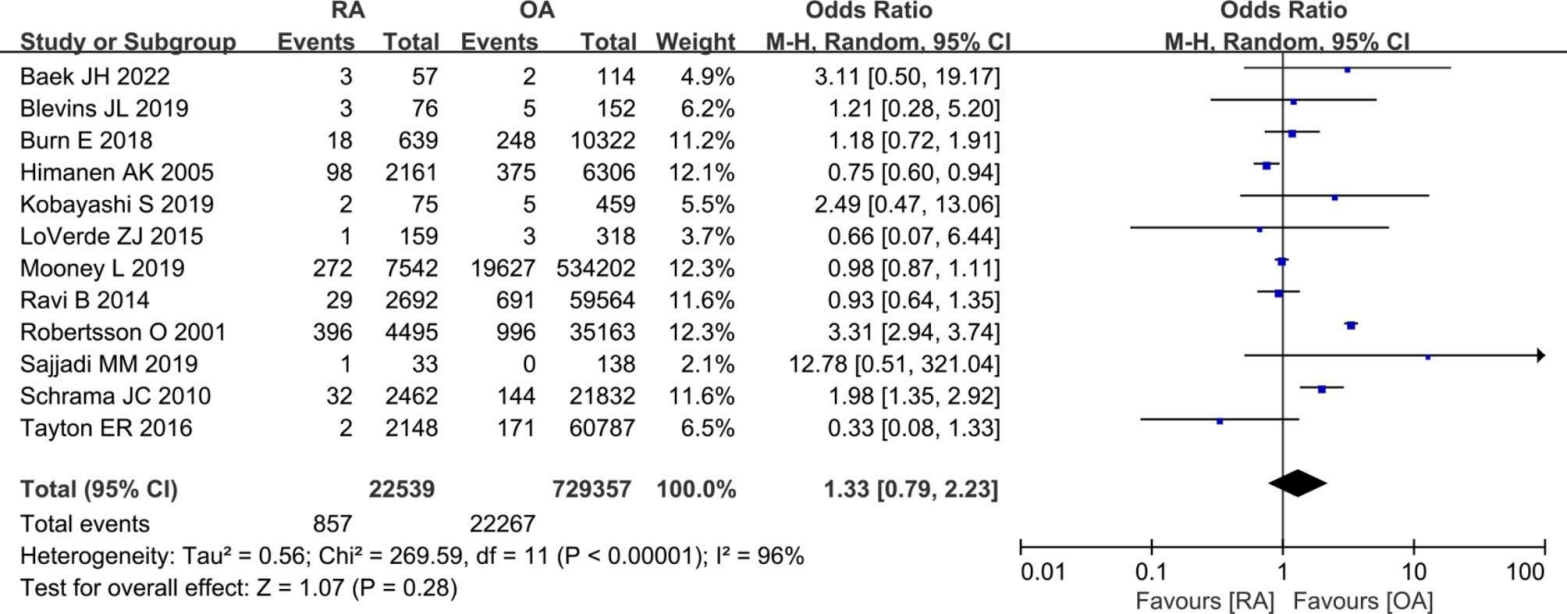
Forest plot showing the revision rate after primary TKA in patients with RA versus patients with osteoarthritis OA

We performed a meta-analysis comparing the revision rate in patients with RA versus OA including twelve studies [9, 11, 18, 21, 23, 27, 30, 31, 34, 35, 37, 39]. The rate of revision after primary TKA was no statistical difference between RA patients and OA groups (857/22,539 vs. 22,267/729,357; OR 1.33, [95% CI 0.79–2.23]; *I*^2^ = 96%; *P* = 0.28) (Fig. 4).

#### VTE

**Fig. 5:**
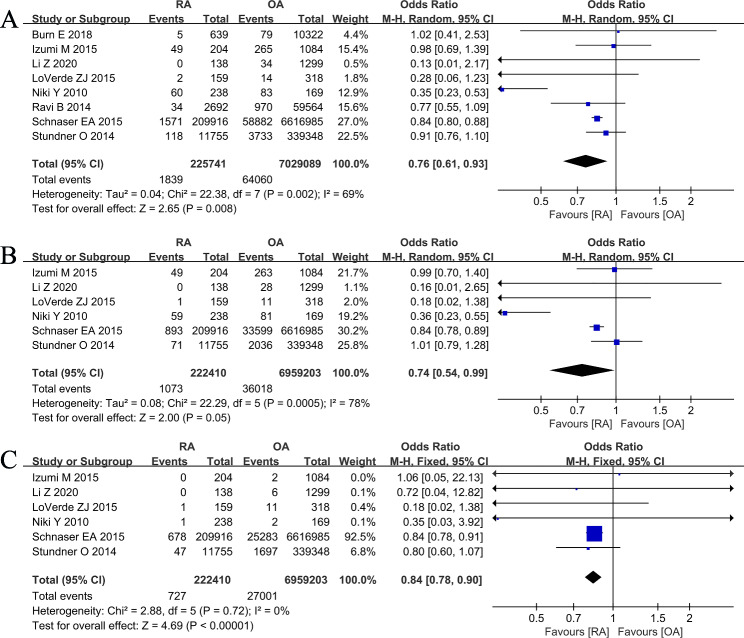
Forest plot showing the likelihood of venous thromboembolism after primary TKA in patients with RA versus patients with osteoarthritis OA. (**A**). venous thromboembolism (VTE, DVT + PE) (**B**). deep venous thrombosis (DVT) (**C**). pulmonary embolism (PE)

A total of eight studies [8, 23, 28, 31, 32, 34, 36, 38] provided data comparing the VTE rate in patients with RA versus OA group. The pooled results showed that the VTE rate of patients with RA was significantly higher than those patients with OA (1839/225,741 vs. 64,060/7,029,089; OR 0.76, [95% CI 0.61–0.93]; *I*^2^ = 69%; *P* = 0.008) (Fig. 5A). We also performed a subgroup analysis based on DVT and PE. Subgroup analysis of six studies [8, 28, 31, 32, 36, 38]reported that the rate of DVT after TKA was slightly higher in RA group than that in OA group (1073/222,410 vs. 36,018/6,959,203; OR 0.74, [95% CI 0.54–0.99]; *I*^2^ = 78%; *P* = 0.05) (Fig. 5B), and PE rate in patients with RA were significantly higher than OA patients (727/222,410 vs. 27,001/6,959,203; OR 0.84, [95% CI 0.78–0.90]; *I*^2^ = 0%; *P*<0.00001) (Fig. 5C).

#### Mortality

**Fig. 6:**
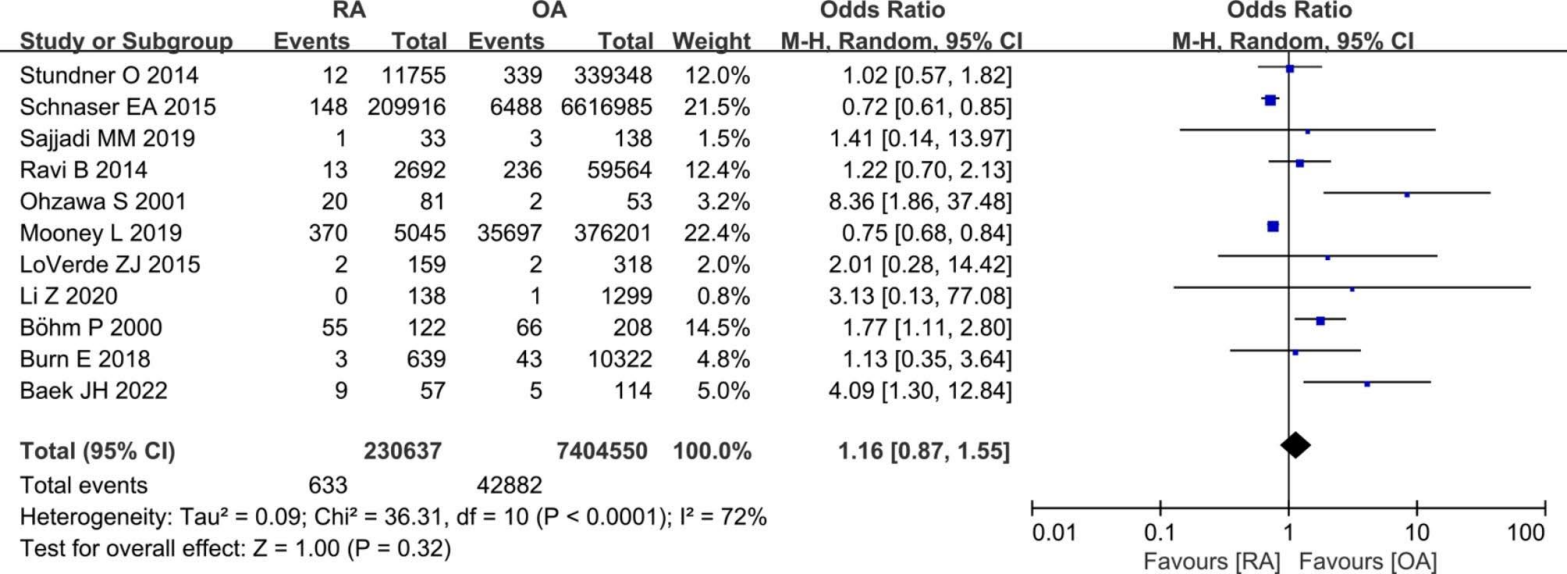
Forest plot showing mortality rate after primary TKA in patients with RA versus patients with osteoarthritis OA

Eleven studies [8, 9, 11, 18, 22, 23, 31, 33, 34, 36, 38] reported mortality between RA patients and OA patients. The pooled results showed that there were no statistically significant differences in rate of mortality between the two groups (633/230,637 vs. 42,882/7,404,550; OR 1.16, [95% CI 0.87–1.55]; *I*^2^ = 72%; *P* = 0.32) (Fig. 6).

### Periprosthetic fractures

**Fig. 7:**
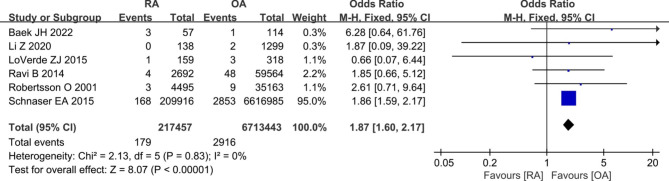
Forest plot showing the likelihood of periprosthetic fractures after primary TKA in patients with RA versus patients with osteoarthritis OA

A total of six studies [8, 11, 31, 34–36] provided data comparing the periprosthetic fractures rate in patients with RA versus OA group. The meta-analysis revealed that patients with RA dramatically increased risk of periprosthetic fractures compared to patients with OA (179/217,457 vs. 2916/6,713,443; OR 1.87, [95% CI 1.60–2.17]; *I*^2^ = 0%; *P*<0.00001) (Fig. 7).

### Prosthetic loosening and length of stay

**Fig. 8:**
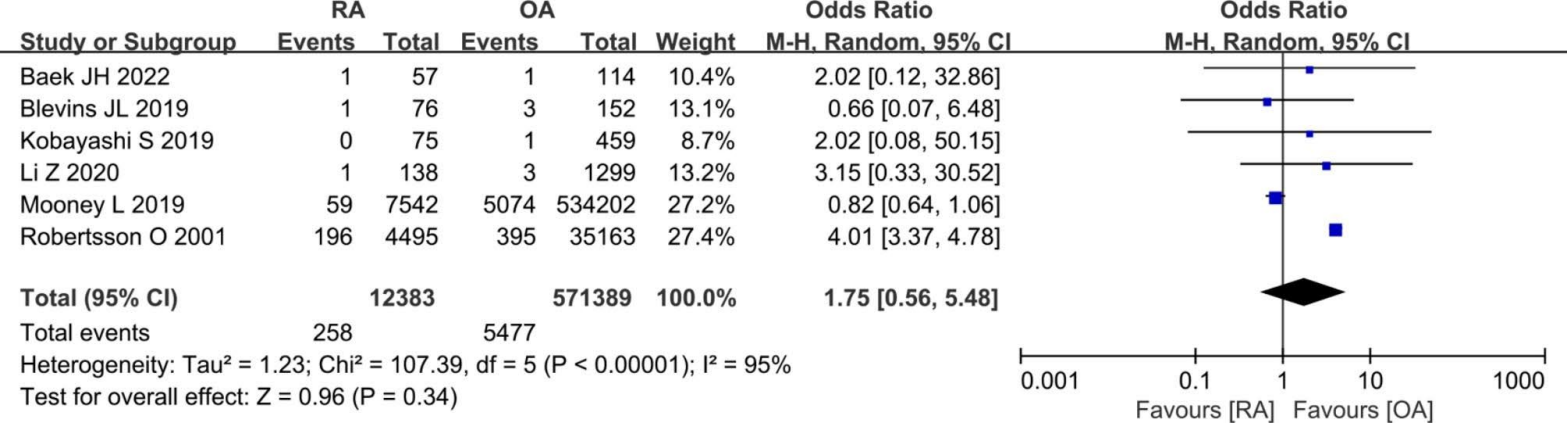
Forest plot showing the likelihood of prosthetic loosening after primary TKA in patients with RA versus patients with osteoarthritis OA

**Fig. 9:**
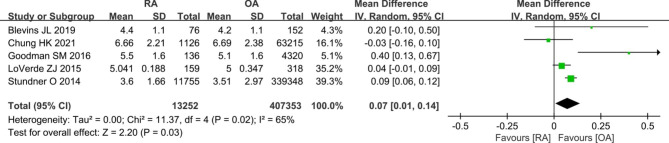
Forest plot showing length of stay underwent primary TKA in patients with RA versus patients with osteoarthritis OA

Six studies [8, 9, 11, 21, 30, 35] provided data comparing the prosthetic loosening rate in patients with RA versus OA group. The meta-analysis demonstrated that patients with RA were no statistically significant difference compared to OA (258/12,383 vs. 5477/571,389; OR 1.75, [95% CI 0.56–5.48]; *I*^2^ = 95%; *P* = 0.34) (Fig. 8). And five studies [7, 21, 26, 31, 38] reported length of stay between RA patients and OA patients. The pooled results showed that patients with RA had long length of stay compared with OA patients (WMD 0.07, [95% CI 0.01–0.14]; *I*^2^ = 65%; *P* = 0.03) (Fig. 9).

## Discussion

In this review of 24 studies, we found strong evidence for increased risk of overall infection, deep infection, VTE, PE and periprosthetic fractures, and reasonable evidence for increased risk of DVT and length of stay after TKA in patients with RA versus OA. Meanwhile, the results demonstrated that no evidence to support any differences in superficial site infection (SSI), revision rate, mortality and prosthetic loosening following TKA in patients with RA versus OA. However, it surprised that in patients with RA achieve higher satisfaction compared to patients with OA, which was conversed as we hypothesized. According to a previous meta-analysis by Ravi et al [19], patients with RA have a higher risk of infection after TKA than those with OA, but they observed no differences regarding revision, mortality, or VTE. Our meta-analysis included a larger simple size, more recent data, and more outcome measures, The current study reported that studies published from January 1, 2000 to October 15, 2022 (to more closely reflect current clinical practice) of primary TKA and contained information on outcomes in RA and OA patients. We systematically collected relevant clinical trials of patients with RA and OA undergoing TKA and performed a meta-analysis and systematic review in this study.

Our present study revealed that patients with RA with the similar rate in superficial site infection, but much higher rate in deep and overall infection rate compared to OA patients. TKA is used to alleviate pain and improve mobility extensively in patients who develop severe destructive changes of their knee joints due to inflammatory or degenerative musculoskeletal diseases [16]. Of all the complications, prosthetic joint infection (PJI) is the most devastating in elective orthopaedic surgery, the incidence of PJI after TKA has been reported to be approximately 1–2% [40, 41]. The patient occurred PJI will usually be removal or exchange of the prosthesis associated with poor functional, long hospital stay, prolonged use of antibiotics and higher resource consumption burdens [40]. Previous studies have reported conflicting results concerning the risk of PJI after TKA for RA and OA [7, 11, 23, 31]. Two previous meta-analysis have revealed that compared with OA, patients with RA are at higher risk of infections after TKA [19, 42], in line with those studies, we also found an increased risk of postoperative infection among patients with RA, but similar rate in superficial site infection. The Guidelines revealed that the risk of postoperative infection complications after total joint arthroplasty (TJA) was increased in patients with RA nearly 2-fold, and deep infection complications increased by 1.5-fold [43]. Yeganeh et al. demonstrated that PJI among patients with RA following TKA is 1.6-fold greater than in patients for OA [44]. A cohort study with 71,793 patients reported that RA are at higher risk of infection after TKA relative to those with OA (1.26%, compared with 0.84% for recipients with OA [34]. Furthermore, a retrospective study with large samples also proved that revision for infection was significantly higher in the RA (HR = 1.37 (1.11–1.69), *P* = 0.003) compared to OA [9]. On the contrary, Chung et al. found no significant difference in acute TKA surgical site infection risk between RA and OA patients when controlling for potential confounders [7], in line with our study, similar rate surgical site infection may due to standard antirheumatic therapy and welled perioperative management. While da Cunha et al. also considered RA was not identified as a risk factor for perioperative infections in TKA [25]. This higher risk may be due to the immunosuppressive therapies for RA patients including disease-modifying antirheumatic drugs (DMARDs), corticosteroids [45–47], 46% of RA patients were receiving biologic DMARDs, 67% were receiving nonbiologic DMARDs, and 25% were receiving glucocorticoids [43]. On the other hand, RA patients are more susceptible to postoperative anemia and are more likely to require a blood transfusion because of bone marrow suppression with chronic disease or medication use, while blood transfusions may increase the risk of infection [7]. In addition, vulnerable soft tissue envelope around the knee joint could make the TKA in RA patients more susceptible to infection [37]. Therefore, preoperative management of those patients is well prepared and perioperative adverse events may decrease and during the perioperative period, anti-rheumatic therapy should be more standard to avoid infection in RA patients [10, 43].

Our meta-analysis demonstrated that there were no significant differences in the revision rate between RA patients and OA patients. It is reported that lots of revision TKA procedures continue increasing at a high rate with the number of TKA rising, while infection and aseptic loosening were the two most common reasons for revision in both OA and RA following TKA [48, 49]. Theoretically, RA patients have a higher infection rate, so the revision rate should be higher. The McMaster Arthroplasty Collaborative (MAC) found that 1.41% of individuals experienced revision TKA for PJI in 2022 [50]. Because less number of TKA for RA compared to OA, so a small part of these revision procedures are performed in RA patients. Revision TKA in patients with RA will be very challenging due to medical comorbidities, poor bone stock and soft tissue, substantially increases the risk of postoperative complications. Several previous studies have compared revision rate after TKA in patients with RA and OA patients. A Prospective, Population-Based Study with 24,293 patients (2,462 knees in the RA 21,832 knees in OA) proved that RA patients had a higher risk of revision(RR 5.4, 95% CI 1.9–16; *P* = 0.002), while had a 1.6 times higher risk of revision for infection after TKA(RR 4.1, 95%CI 1.6–11; *P* = 0.004) versus OA patients [37]. Another retrospective study revealed that patients with RA had a significantly increased risk of overall revision TKA(RR 1.6, 95% CI 1.5–1.6; *P*<0.0001) compared with patients with OA. On the contrary, Burn et al. stated that there was no significant in the incidence of revision over the 10 years following TKA among individuals with RA compared to those with OA [23]. Meanwhile, by using Kaplan–Meier survivorship analysis, Abram et al. estimated the revision rate without statistical difference in RA patients compared with OA patients during long-term follow-up [51]. Interestingly, a large national database proved that the rate of revision after TKA in RA patients is lower than those with OA [9]. Consequently, the higher revision rate in RA patients may be related to their younger age at the time of surgery or high risk of infection, while similar or lower revision rate due to comorbidities and weak bone stocks of RA that surgeons may preferentially conservative treatment, such as conducted knee infections with antibiotics or debridement instead of revision, and decreased wear of the prosthesis due to lower physical activity in patients with RA.

VTE including DVT and PE are the most dreadful and potentially life threatening complications after TKA and other orthopedic surgical procedures, because of its closely linked with mortality and health care costs [32]. Previous studies have demonstrated that patients who have significantly higher risk of VTE after lower extremity surgery, especially RA patients following TKA [34, 36]. However, although the implementation of greatly enhanced antithrombotic prevention, the incidence of VTE after TKA remains high [8]. But whether RA is a potential candidate predisposing patients to postoperative VTE have demonstrated highly variable outcomes. Several studies have reported on differences in perioperative outcomes between RA and OA patients performing TKA. A retrospective study using National Surgical Quality Improvement Program (NSQIP) database by Jauregui et al. revealed that no significant difference was found in the incidence of PE (*P* = 0.99), and DVT (*P* = 0.72) [52]. Another study contains 355 patients (238 knees in the RA, 169 knees in OA) demonstrated that the incidence of DVT after TKA was significantly higher in OA patients than in those with RA, interestingly, when the patients were adjusted for age and anti-inflammatory drugs (NSAIDs) use, the incidence of DVT was similar in the two groups [32]. Therefore, RA may not represent a predictor for VTE according to their research. But some studies have demonstrated a higher rate of VTE in patients with RA undergoing TKA compared to OA patients [8, 31], and hypercoagulability with reduced fibrinolysis owing to raised levels of autoantibodies and vascular endothelium is easily damaged might illustrate these findings. Furthermore, frequent use of NSAIDs with the resulting antiplatelet activity and RA patients along with younger age distribution, and lower body mass index (BMI), which advocating a lower thrombotic risk [32]. We also speculated that the lower hemoglobin and blood dilution may also be the reason for the similar or less incidence of thrombosis in RA patients. In our present study, RA patients was associated with significant differences in the risk for VTE following TKA, so surgeons should pay more close attention to the thromboprophylaxis use of anticoagulant therapies and perioperative drug management after TKA in patients with RA.

Our meta-analysis, however, found no significant difference in rate of mortality between RA and OA patients. It is generally considered that patients suffering from RA were shown to have higher rates of mortality after TKA due to higher rates of infection, cardiac morbidity, VTE and pulmonary disease [48]. Most surgeons emphasize 30-day and 90-day mortality for RA patients following TKA, because the increased mortality rates in years 1–10 suggest disease-specific rather than surgery-induced mortality [9, 38]. Surprisingly, most of the literatures available on mortality of patients undergoing TKA for RA patients support that there is no significant difference in mortality compared to OA patients. For example, Domsic et al. revealed that RA patients had an OR of 0.771 (95%CI 0.570–1.041, *P* = 0.09) for mortality with TKA versus OA patients at the final follow-up(10years) by using data from the Nationwide Inpatient Sample (1993–2006) [53]. Similarly, a present study including more than 7 million patients undergoing different types of surgery including TKA found no differences in the incidence of mortality among patients with a diagnosis of RA compared to those without [54]. But a study by Baek et al. demonstrated that the overall mortality rates in the RA and OA groups were 15.8% (9/57) and 4.4% (5/114) at the final follow-up(10years), respectively (*P* < 0.05) [11], and Michaud et al. also revealed that RA was associated with a significantly higher long-term mortality [55]. Mortality rates following TKA are reported to be high in patients with RA, which may be due to surgical techniques, prostheses, non-standard anti-rheumatoid therapy, and improper perioperative management. So, orthopedic surgeons take greater perioperative management and long-term postoperative follow-up when operating on RA patients will improve the survival of RA patients.

The interesting finding of this study was that TKA in patients with RA showed higher rates of post-operative periprosthetic fractures when compared to OA. To our knowledge, there is no relevant meta-analysis to analyze and summarize periprosthetic fractures. However, with increasing percentages of TKA worldwide, it is likely that the absolute burden of periprosthetic fractures will continue to grow, which along with worse functional outcomes and higher medical costs [56]. Schnaser et al. reported that patients with RA had significantly more inpatient post-operative periprosthetic fractures when compared to OA (*P*<0.01) [36], while Abram et al. also reported that there were more periprosthetic fractures in the RA patients than OA patients and all occurred more than 5 years after TKA [51]. RA patients usually are more prone to osteoporosis due to chronic steroid treatment and physical impairment, which all can result in osteopenia and related fractures. Therefore, surgeons should be aware that RA patients are possibly to be at risk of periprosthetic fracture. Another finding of our meta-analysis was that RA patients had similar incidence of loosening after TKA compared with OA patients. Loosening is the major cause of revision among various complications, and it tends to occur many years after the initial surgery, survival of primary TKA is substantially diminished with each consecutive revision [57, 58]. Therefore, the early detection of loosening in patients with TKA has become a dominating importance and interest in the orthopedic field. Most of previous literatures showed that patients with RA had no significant difference in loosening rate than patients with OA probably because decreased wear of the prosthesis due to lower physical activity in patients with RA [9, 11, 30]. In conclusion, rigorous control of RA activity by biological treatment not only controls local joint inflammation, which would improve bone quality and reduced rate of premature loosening of the implant.

In our present study RA patients had longer mean lengths of hospitalization with an OR of 0.07 (95%CI 0.01–0.14, *P* = 0.03) compared to OA patients, while several studies have also shown that RA patients would prolong hospitalization [21, 26, 38]. The hospitalization after TKA depends on a variety of factors, including the patient’s health status, ability to walk safely, overall pain control, and the physical activity in patients with RA. Longer hospitalization increased infection rates and health care costs, therefore, surgeons must pay more attention to perioperative management of patients with RA and shorten the length of stay. Currently, patient satisfaction gains attention as an important outcome measure, because there is a well-documented discrepancy between clinician and patient ratings of health status. Furthermore, better postoperative analgesia, ambulation, patient symptom, expectation, minimal drug consumption and complications all correlated with patient satisfaction. But satisfaction is evaluated in different ways in previous studies that cannot be pooled analysis, which RA patients have similar or more excellent outcomes after TKA compared with OA patients [18, 26, 30, 59]. This may be related to the fact that the preoperative symptoms of RA are more severe, and that patients with RA recovery faster with younger age and have lower expectations.

Some limitations must be taken into consideration when interpreting the results of this study. Firstly, there have no elaborate information on other potential confounders, such as length of surgery time, blood loss, medication use, disease activity or other non-measurable factors (e.g., the types of implants, surgical technique, surgical approach, etc.). Further research is necessary to elucidate for these findings, including anti-rheumatic medication use, implant choice, perioperative antibiotic prophylaxis, and method of rehabilitation following TKA. Secondly, the diagnostic criteria for RA are different, the timing of the diagnosis of PJI is different, deep or superficial infection is not clear in some studies, such a high rate of misclassification may threaten our study and we cannot analyze these risk factors or outcomes in this study. Thirdly, some of the studies more than 10 years ago, surgical techniques and medicine may be different from those currently used. As many outcomes were included in a small number of literatures, we did not perform subgroup analyses. More studies are needed to perform subgroup analyses of these outcomes. Finally, the studies included are mainly retrospective case-control studies and cohort studies, with no randomized controlled trials studies, so more prospective studies and confounders controlled are warranted to evaluate short-term and long-term clinical outcomes of TKA in patients with RA. These recognized limitations are inherent to all studies using this database design and could potentially be improved through prospective data collection.

To our knowledge, this is the first study that has compared outcomes after TKA comprehensively in patients with RA. Our study demonstrated that patients with RA are at higher risk of postoperative infection, VTE, periprosthetic fracture, and lengths of hospitalization compared to patients with OA following TKA, but there are no significant differences in revision rate, mortality and loosening. In addition, we also should be aware of the possibility that RA patients may have an increased risk for perioperative transfusions, an increased incidence of pneumonia and cardiac complications, surgeons must pay more attention to perioperative management of patients with RA and dramatically reduce the risk of complications. In conclusion, despite RA increased incidence of postoperative infection, VTE, periprosthetic fracture, and lengths of hospitalization, TKA should continue to be presented as an effective surgical procedure for patients whose conditions are intractable to conservative and medical management of RA.

## Electronic supplementary material

Below is the link to the electronic supplementary material.

Supplementary Material 1

## Data Availability

All data generated or analyzed during this study are included in the Additional file.

## Abbreviations

TKA: Total knee arthroplasty
RA: Rheumatoid arthritis
OA: Osteoarthritis
OR: Odds ratio
M-H: Mantel-Haenszel
CI: Confidence interval
VTE: Venous thromboembolism
DVT: Deep venous thrombosis
PE: Pulmonary embolism
PPF: Periprosthetic fractures
PL: Prosthetic loosening
LOS: Length of stay
MO: Mortality
WMD: Weighted mean difference
IV: Inverse variance
NOS: Newcastle-Ottawa Scale
CCS: Case-control studies
RCS: Retrospective cohort studies
PCS: Prospective cohort studies
